# Medicaid Coverage Disruptions Among Children Enrolled in North Carolina Medicaid From 2016 to 2018

**DOI:** 10.1001/jamahealthforum.2021.4283

**Published:** 2021-12-23

**Authors:** Rushina Cholera, David Anderson, Sudha R. Raman, Bradley G. Hammill, Bethany DiPrete, Alexander Breskin, Catherine Wiener, Nuvan Rathnayaka, Suzanne Landi, M. Alan Brookhart, Rebecca G. Whitaker, Janet Prvu Bettger, Charlene A. Wong

**Affiliations:** 1 Duke Margolis Center for Health Policy, Durham, North Carolina; 2 Department of Pediatrics, Duke University, Durham, North Carolina; 3 Department of Population Health Sciences, Duke University, Durham, North Carolina; 4 NoviSci, Durham, North Carolina; 5 Injury Prevention Research Center, University of North Carolina at Chapel Hill; 6 Department of Orthopaedic Surgery, Duke University, Durham, North Carolina

## Abstract

**Importance:**

Brief disruptions in insurance coverage among eligible participants are associated with poorer health outcomes for children.

**Objective:**

To describe factors associated with coverage disruptions among children enrolled in North Carolina Medicaid from 2016 to 2018 and estimate the outcome of preventing such disruptions on medical expenditures.

**Design, Setting, and Participants:**

This was a retrospective cohort study using North Carolina Medicaid claims data. All enrolled individuals were aged 1 to 20 years on January 1, 2016, and with 30 days of prior continuous enrollment. Children were observed from January 1, 2016, until December 31, 2018. Analyses were conducted from June 2020 through December 2020.

**Main Outcomes and Measures:**

Risk of Medicaid coverage disruptions of 1 to less than 12 months was assessed. Among children who disenrolled from Medicaid for 30 or more days, the risk of reenrollment within 1 to 6 months and 7 to 11 months was assessed. An inverse probability of censoring weights method was then used to estimate the outcome of an intervention to reduce coverage disruptions through preventing disenrollment on per member per month (PMPM) cost.

**Results:**

The study population included 831 173 Medicaid beneficiaries aged 1 to 5 years (23%), 6 to 17 years (68%), and 18 to 20 years (9%); 35% were Black, 44% were White, and 14% were Hispanic/Latinx. Among those with a first disenrollment (n = 214 401, 26%), the risk of reenrollment within 6 months and 7 to 11 months was 19% and 7%, respectively. Risk of coverage disruption was higher for Black children (hazard ratio [HR], 1.21; 95% CI, 1.18-1.24), children of other races (Asian, American Indian, Hawaiian or Pacific Islander, multiple races, or unreported; HR, 1.37; 95% CI, 1.33-1.40), and Latinx children (HR, 1.65; 95% CI, 1.60-1.70) compared with White children. Risk of coverage disruption was also higher for children with higher medical complexity (HR, 1.15; 95% CI, 1.12-1.19). The risk of coverage disruption was lower for children living in counties with the highest unemployment rates (HR, 0.89; 95% CI, 0.85-0.94), and comparisons between county-level measures of child poverty and graduation rates showed little or no difference. The estimated PMPM cost for the full population under a scenario in which all medical costs were included was $125.73. Estimated PMPM cost for the full cohort in a counterfactual scenario in which disenrollment was prevented was slightly lower ($122.14). Across all subgroups, estimated PMPM costs were modestly lower ($2-$8) in the scenario in which disenrollment was prevented.

**Conclusions and Relevance:**

In this cohort study, the risk of Medicaid coverage disruption was high, with many eligible children in historically marginalized communities continuing to experience unstable enrollment. In addition to improving health outcomes, preventing coverage gaps through policies that decrease disenrollment may also reduce Medicaid costs.

## Key Points

**Question:**

Which populations of children are at risk for Medicaid coverage disruptions, and what is the outcome of preventing disenrollment on medical expenditures?

**Findings:**

Among 831 173 Medicaid-enrolled children from 2016 to 2018, more than 25% who disenrolled experienced coverage disruptions, with the highest risk observed among Latinx children. A hypothetical intervention to prevent disenrollment was associated with modest decreases in cost.

**Meaning:**

Large proportions of Medicaid-enrolled children continue to experience preventable coverage gaps that can have long-term negative health consequences and may lead to increased medical expenditures; policies to address barriers to Medicaid retention among children at highest risk of coverage disruptions are needed.

## Teaser

This cohort study describes factors associated with coverage disruptions among children enrolled in North Carolina Medicaid from 2016 to 2018 and estimates the outcome of preventing such disruptions with medical expenditures.

## Introduction

Disruptions in insurance coverage are associated with reduced health care access and unmet health care needs for children.^1,2,3,4^ Children with even short periods of uninsurance experience delays in care, are less likely to receive preventive care, and are more likely to seek emergency care for ambulatory conditions.^5,6,7,8^ Policy changes following the Children’s Health Insurance Program Reauthorization Act of 2009 and the Affordable Care Act (ACA) in 2014 improved enrollment and retention in Medicaid among children. However, in recent years, enrollment in Medicaid and the Children’s Health Insurance Program (CHIP) decreased by more than a million children, partly thought to be due to state-level policies that increase coverage disruptions for eligible children.^9,10^ More than 30% of children eligible for Medicaid or CHIP still experience insurance coverage gaps yearly, with substantial variation among states.^11,12,13^ Importantly, insurance coverage gaps are more common among children living in rural areas and those experiencing social risks, including lower income, housing instability, or fears of immigration enforcement.^5,7,11,14,15,16,17^

Brief health insurance coverage gaps characterized by disenrollment followed by reenrollment within a short time period (“churn” in the health policy literature) are often secondary to administrative barriers during the reenrollment process, though other reasons include changes in family circumstances, employment, or switching insurance coverage.^9,18^ In addition to negatively affecting health, this unstable enrollment can also lead to increased health care spending because of programmatic inefficiencies and increased administrative burden.^19^ Pre-ACA estimates show that average monthly medical expenses for children enrolled in Medicaid over the course of a year decrease the longer they are enrolled in Medicaid.^20^ Medical cost savings with longer enrollment are thought to occur for 2 primary reasons: (1) longer enrollment may allow children to receive preventive care, thus reducing utilization of expensive acute care, and (2) people may enroll in Medicaid when they are sick, leading to higher medical expenditures soon after enrollment.^20^

The broad adoption of Medicaid managed care includes transitions that risk increasing unstable enrollment, both within Medicaid and among managed care plans.^21^ In July 2021, North Carolina Medicaid transitioned from a traditional single-payer fee-for-service model to a managed care structure with multiple plans, necessitating careful monitoring of Medicaid enrollment patterns in this non–Medicaid expansion state. In this context, we sought to establish a baseline risk for coverage disruptions among children enrolled in North Carolina Medicaid from 2016 to 2018.^22^ Given limited existing data on the relationship between unstable enrollment and cost of care for children, we also sought to estimate the association between coverage disruptions and medical expenditures by comparing actual costs with a counterfactual scenario in which coverage disruptions were reduced by preventing disenrollment. We hypothesized that the actual cost of care, which includes costs of children who experienced coverage disruptions during the study period, would be higher than the scenario in which disenrollment was prevented.

## Methods

We conducted a retrospective cohort study to describe Medicaid coverage disruptions and to examine the risk of such disruptions across demographic and clinical subgroups of policy relevance among children enrolled in North Carolina Medicaid from 2016 to 2018. We also sought to assess the association between disenrollment and per member per month (PMPM) cost. Analyses were conducted from June 2020 through December 2020. This study was determined to be exempt from obtaining informed consent by the Duke University institutional review board owing to the use of deidentified data. This study complies with the Strengthening the Reporting of Observational Studies in Epidemiology (STROBE) reporting guidelines.

### Study Population

North Carolina Medicaid claims data from January 1, 2016, through December 31, 2018, were used for these analyses. All individuals aged 1 to 20 years on January 1, 2016, and with 30 days of prior continuous enrollment were included. Individuals with non-Medicaid concurrent insurance were excluded because we did not have insight into the continuity or disruption of other insurance sources. Children were observed until the time of aging out at age 21 years, death, or December 31, 2018, whichever occurred first.

### Enrollment Patterns

We examined Medicaid disenrollment and reenrollment patterns throughout the study period. Our primary outcome was Medicaid coverage disruption, defined as disenrollment followed by reenrollment in Medicaid within less than 12 months. Similar to prior work, we examined coverage disruptions of 2 durations, 1 to 6 months and 7 to 11 months, based on the assumptions that (1) brief coverage gaps of less than 1 month were likely due to administrative errors that would not have affected enrollment status, (2) that children who reenrolled in Medicaid within 6 months of coverage loss were likely eligible during the period of disenrollment, and (3) that those who reenrolled within 7 to less than 12 months were possibly eligible for coverage while disenrolled.^13^ To calculate these outcomes, we first assessed the risk of disenrollment, defined as a gap in enrollment of at least 30 days. Among those who disenrolled, we then assessed (1) the risk of first reenrollment, (2) a second disenrollment among those who reenrolled, and (3) a second reenrollment among those who had a second disenrollment. The risk of coverage disruptions was calculated as the risks of reenrollment within 1 to 6 and 7 to 11 months, respectively.

### Cost

All costs in the claims data within the study period were included (ie, institutional, professional, and pharmacy claims). The PMPM cost was estimated.

### Covariates

To describe groups at increased risk for Medicaid coverage disruptions, we described the dynamics of enrollment overall and by subgroups defined by age, sex, race, ethnicity, and medical complexity. Demographic characteristics including race and ethnicity were extracted directly from Medicaid claims. Medical complexity was defined by the Pediatric Medical Complexity Algorithm (PMCA), which assigns children into 1 of 3 categories: (1) children without chronic disease (“without CD”), (2) children with noncomplex chronic disease (“NC-CD”), or (3) children with complex chronic disease (“C-CD”).^23^ Given the potential for coverage disruptions to be associated with geography and social risk factors, we also examined community-level factors, including^24^: rural/urban residence, graduation rate, child poverty rate, and unemployment rate, categorizing graduation rate, child poverty rate, and unemployment rate by quartiles across the 100 counties in North Carolina.

### Statistical Analysis

We first estimated the proportion of potential member months, defined as the sum across individuals, of the maximum number of months an individual could be enrolled in the study (ie, 36 months), during which individuals were actually enrolled. To estimate the risk of a disenrollment and subsequent reenrollment, we used an estimating function representation of the Kaplan-Meier estimator. Risk of disenrollment was estimated from the index date until end of follow-up. Among those who disenrolled, the risk of reenrollment was estimated from the first day of disenrollment (ie, 31 days after last enrollment day) until the end of follow-up. We followed a similar process to estimate the risk of a second disenrollment and a second reenrollment, starting with the date of first reenrollment and the date of second disenrollment, respectively. Analyses were conducted within the covariate subgroups outlined previously. To identify characteristics associated with coverage disruptions, we fit a Cox proportional hazards model to estimate hazard ratios (HRs) and 95% CIs in the population of individuals who experienced a first disenrollment. Covariates included all characteristics outlined previously. Death and aging out of the cohort were treated as competing risks in all analyses.

We sought to estimate the outcome of preventing disenrollment on average PMPM cost (eAppendix in the Supplement). The PMPM cost was estimated by computing the average cost in each month over the 36 months and then taking the average of the average monthly costs. Cost was estimated under the following 3 scenarios:

1. The natural course estimator included all costs accrued across any period of enrollment, including the PMPM costs seen during enrolled periods for patients who experienced coverage disruptions.
2. The unadjusted estimator included costs up to the first disenrollment. This means that for patients who remain enrolled the entire time, all costs were included. However, for those who experienced any disenrollment, any costs accrued during subsequent periods of reenrollment were discarded.
3. The counterfactual estimator included costs up to the first disenrollment, ie, the same costs as the unadjusted estimator. A modified version of the Bang and Tsiatis censored cost estimator that uses inverse probability of censoring weights was used to provide an estimate of the counterfactual PMPM cost if no disenrollment occurred.^25^ Weights were estimated using a logistic regression model with a set of covariates assumed to achieve conditional exchangeability between those who were censored owing to disenrollment and those who were uncensored.

The counterfactual estimator was designed to reflect the PMPM costs that would have accrued over the full 36-month time period if patients who disenrolled under the natural course had been prevented from disenrolling altogether. Because weights were used to make the beneficiaries who remained enrolled stand in for those who disenrolled, the counterfactual estimator can be interpreted as what would happen if no one had experienced a disenrollment with the conditional exchangeability assumptions described previously. For example, under the hypothesis that coverage disruptions might be associated with cost through selective enrollment during expensive health care episodes, such higher costs would be reflected in the natural course scenario but not in the counterfactual scenario.

Before modeling, all costs were aggregated to the level of week. For the total estimates of all individuals in a given cohort, the logistic regression model used to estimate the weights modeled the disenrollment outcome with the following covariates: week, baseline age, PMCA category at baseline, lagged average weekly cost, and cost accrued in the previous month. Categorical variables were modeled with binary indicator variables. The continuous variable week was modeled with a restricted cubic regression spline with 5 knots to allow a very flexible model for the time trend. The continuous variables of lagged average weekly cost and cost accrued in the past month were modeled with restricted cubic regression splines with 3 knots. A log-rank test was used for statistical comparisons; 2-sided *P* < .05 was used to indicate statistical significance. All analyses were performed using R, version 3.5.2 (R Foundation for Statistical Computing).^26^

## Results

### Population Characteristics

More than 1.6 million children were enrolled in North Carolina Medicaid on January 1, 2016. After exclusion criteria were applied, the study population included 831 173 children (Table 1). The age distribution was 1 to 5 years (23%), 6 to 17 years (68%), and 18 to 20 years (9%); 35% were Black, 44% were White, and 14% were Hispanic/Latinx. Beneficiaries were overwhelmingly urban residents (74%).

**Table 1.** Cumulative Incidence of Medicaid Disenrollment and Reenrollment by Child Characteristics

Characteristic	Individuals, % (95% CI)					
	Disenrollment		Reenrollment			
	No. at risk	Risk of disenrollment	No. at risk	Risk of reenrollment, mo		No reenrollment
				1-6	7-11	
Overall	831 173	25.8 (25.8-25.8)	214 401	18.7 (18.7-18.7)	7.3 (7.3-7.3)	74.0 (74.0-74.0)
Age, y						
1-5	189 569	24.9 (24.9-24.9)	47 199	20.6 (20.6-20.6)	8.5 (8.5-8.5)	70.8 (70.8-70.8)
6-11	302 458	23.2 (23.2-23.2)	70 105	20.6 (20.6-20.7)	8.6 (8.6-8.6)	70.8 (70.8-70.8)
12-17	261 209	29.1 (29.1-29.1)	75 997	17.6 (17.6-17.6)	7.5 (7.5-7.5)	74.9 (74.9-74.9)
18-20	77 937	27.1 (27.1-27.1)	21 100	12.0 (12.0-12.0)	4.4 (4.4-4.4)	83.5 (83.5-83.5)
Sex						
Female	410 306	25.3 (25.3-25.3)	103 690	20.1 (20.1-20.1)	8.2 (8.2-8.2)	71.6 (71.6-71.7)
Male	420 867	26.3 (26.3-26.3)	110 711	17.3 (17.3-17.3)	7.2 (7.2-7.2)	75.5 (75.5-75.5)
Race						
Black	287 069	22.4 (22.4-22.4)	64 247	18.2 (18.2-18.2)	7.7 (7.7-7.7)	74.1 (74.0-74.1)
White	368 402	28.8 (28.8-28.8)	106 268	16.4 (16.4-16.4)	7.1 (7.1-7.1)	76.4 (76.4-76.4)
Other/unknown^a^	175 702	25.0 (25.0-25.0)	43 886	24.8 (24.8-24.8)	9.1 (9.1-9.1)	66.1 (66.1-66.1)
Ethnicity						
Hispanic	113 263	25.1 (25.1-25.1)	28 401	29.5 (29.5-29.5)	9.9 (9.8-9.9)	60.6 (60.6-60.6)
Not Hispanic	710 938	25.9 (25.9-25.9)	183 789	17.2 (17.2-17.2)	7.5 (7.5-7.5)	75.4 (75.4-75.4)
Missing	6972	31.7 (31.7-31.7)	2211	4.6 (4.6-4.6)	2.2 (2.2-2.2)	93.3 (93.2-93.3)
Geography						
Appalachian	136 776	27.1 (27.1-27.1)	37 051	18.5 (18.5-18.5)	7.8 (7.8-7.8)	73.6 (73.6-73.7)
Urban	616 413	26.6 (26.6-26.6)	164 050	18.9 (18.9-18.9)	7.6 (7.6-7.6)	73.5 (73.5-73.5)
Rural	210 428	23.0 (23.0-23.0)	48 318	18.3 (18.3-18.3)	8.0 (8.0-8.0)	73.7 (73.7-73.7)
Missing	4332	46.9 (46.9-46.9)	2033	9.4 (9.4-9.4)	6.4 (6.4-6.4)	84.2 (84.2-84.2)
PMCA^b^						
1 Healthy	554 031	28.4 (28.4-28.4)	157 509	18.4 (18.4-18.4)	7.7 (7.7-7.7)	73.9 (73.9-73.9)
2 NC-CD	138 941	21.2 (21.2-21.2)	29 393	19.4 (19.4-19.4)	7.8 (7.8-7.8)	72.8 (72.8-72.8)
3 C-CD	138 201	19.9 (19.9-19.9)	27 499	19.7 (19.7-19.7)	7.6 (7.6-7.6)	72.7 (72.7-72.8)

Abbreviations: C-CD, complex chronic disease; NC-CD, noncomplex chronic disease; PMCA, Pediatric Medical Complexity Algorithm.

^a^ Other/unknown race includes Asian, American Indian, Hawaiian or Pacific Islander, multiple races, and unreported.

^b^ PMCA categories: 1: healthy, without chronic disease; 2: noncomplex chronic disease; 3: complex chronic disease.

### Risk of Medicaid Coverage Disruption

**Figure 1.**
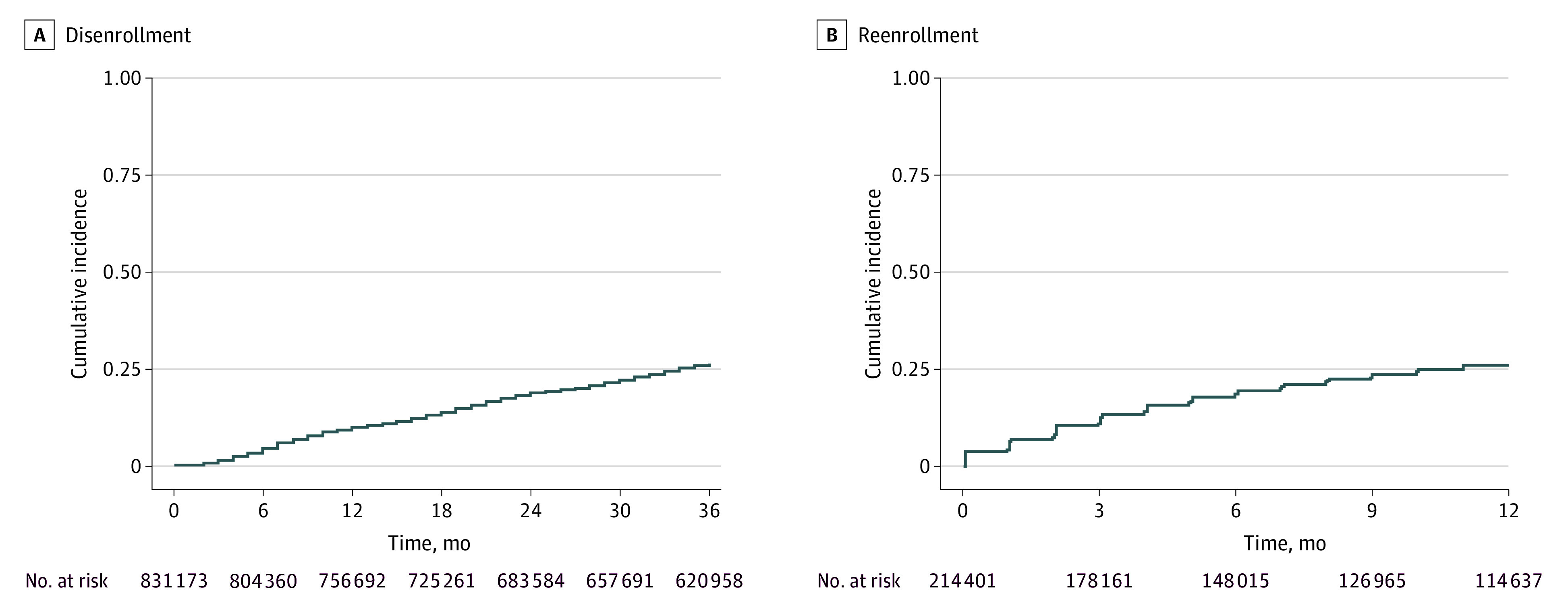
Cumulative Incidence of Disenrollment and Reenrollment From Medicaid A, Cumulative incidence of a first Medicaid disenrollment event among all enrolled children. B, Cumulative incidence of a reenrollment event within 1 year among those who experienced a first disenrollment during the study period.

The risk of experiencing a first disenrollment during the 3-year study period was 26%. Among those with a first disenrollment (n = 214 401), the risk of reenrollment within 1 to 6 months was 19%, and the risk of reenrollment within 7 to 11 months was 7%. The remaining 74% of children remained disenrolled. Of those who experienced a coverage disruption (n = 59 098), the risk of a second disenrollment during the study period was 38%. Among those with a second disenrollment (n = 10 511), the risk of a second episode of reenrollment within 1 to 6 months was 30%, and the risk of reenrollment within 7 to 11 months was 8%, while 62% of children remained disenrolled (Table 1, Figure 1). The risk of a second coverage disruption within the 3-year study period was about 11% for children who experienced a first coverage disruption.

### Bivariate Analyses

**Figure 2.**
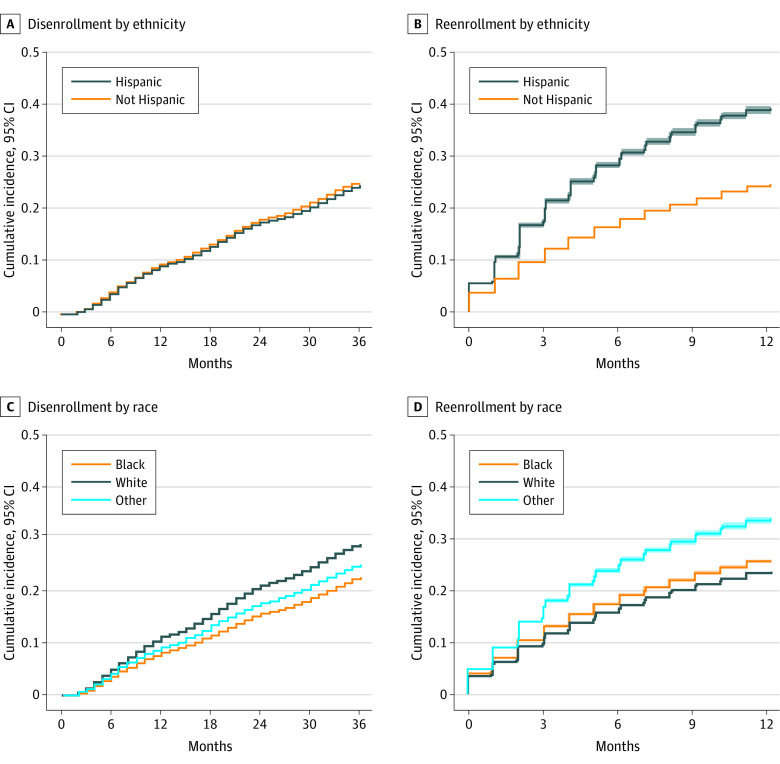
Cumulative Incidence of Medicaid Coverage Disruption Stratified by Ethnicity and Race A, Cumulative incidence of first disenrollment among all enrolled stratified by ethnicity. B, Cumulative incidence of a reenrollment event within 1 year among those who experienced a disenrollment stratified by ethnicity. C, Cumulative incidence of first disenrollment among all enrolled stratified by race. D, Cumulative incidence of a reenrollment event within 1 year among those who experienced a disenrollment stratified by race. Other race includes Asian, American Indian, Hawaiian or Pacific Islander, multiple races, or unreported.

Among those who disenrolled, children aged 1 to 11 years were more likely to reenroll within 6 months (21%) compared with those aged 12 to 17 years (18%) and those aged 18 to 20 years (12%) (Table 1). The risk of a coverage gap of 1 to 6 months was 18% among Black children, 16% among White children, and 25% among children of other races (Asian, American Indian, Hawaiian or Pacific Islander, multiple races, or unreported). Latinx and non-Latinx children had similar risk of disenrollment (25%), but Latinx children had nearly twice the risk of reenrollment within 6 months (30%) compared with non-Latinx children (17%) (Figure 2). Children with the highest medical complexity (C-CD) were at lowest risk for disenrollment (20%) compared with those with NC-CD (21%) and healthy children (28%). Following disenrollment, the risk of reenrollment within 6 months was similar across PMCA categories (18%-20%). While patterns of initial disenrollment differed by geographic and social risk factors, the risk of reenrollment was similar across rural and urban areas and across quartiles of child poverty, unemployment, and graduation.

### Characteristics Associated With Medicaid Coverage Disruptions

A Cox proportional hazards model to identify characteristics associated with coverage gaps of 1 to 6 months showed that older children were less likely to experience these coverage disruptions compared to children younger than 5 years (age 12-17 years: HR, 0.83; 95% CI, 0.81-0.86; age 18-20 years: HR, 0.57, 95% CI, 0.54-0.59) (Table 2). Compared with White children, the risk of a coverage gap of 1 to 6 months was higher for Black children (HR, 1.21; 95% CI, 1.18-1.24) and children of other races (HR, 1.37; 95% CI, 1.33-1.4). Latinx children were also more likely to experience coverage gaps of 1 to 6 months compared with their non-Latinx counterparts (HR, 1.65; 95% CI, 1.60-1.70). Children with higher medical complexity were more likely to experience coverage disruption compared with children with lower complexity (PMCA 2: HR, 1.15; 95% CI, 1.11-1.18; PMCA 3: HR, 1.15; 95% CI, 1.12-1.19). Children living in counties with the highest unemployment rates were less likely to have a coverage gap of 1 to 6 months than those in counties with the lowest quartile unemployment rates (HR, 0.89; 95% CI, 0.85-0.94), and children in counties with the highest graduation rates were slightly less likely to experience coverage disruptions than children in counties with the lowest graduation rates (HR, 0.94; 95% CI, 0.90-0.98). There was no difference in coverage disruptions by county child poverty rate (HR, 1.03; 95% CI, 0.98-1.09).

**Table 2.** Factors Associated With Reenrollment Within 6 Months Among Children Who Experienced a Disenrollment Event in North Carolina Medicaid From 2016 to 2018 (n = 214 401)^a^

Variable	Hazard ratio (95% CI)	*P* value
Age, y		
0-5	1 [Reference]	NA
6-11	0.97 (0.94-1.00)	.02
12-17	0.83 (0.81-0.86)	<.001
18-20	0.57 (0.54-0.59)	<.001
Sex		
Male	1 [Reference]	NA
Female	1.18 (1.15-1.20)	<.001
Race		
Black	1.21 (1.18-1.24)	<.001
White	1 [Reference]	NA
Other/unknown^b^	1.37 (1.33-1.40)	<.001
Ethnicity		
Non-Hispanic	1 [Reference]	NA
Hispanic	1.65 (1.60-1.70)	<.001
Geography		
Urban	1 [Reference]	NA
Rural	1.05 (1.01-1.08)	.004
PMCA		
1 Healthy	1 [Reference]	NA
2 NC-CD	1.15 (1.11-1.18)	<.001
3 C-CD	1.15 (1.12-1.19)	<.001

Abbreviations: C-CD, complex chronic disease; NA, not applicable; NC-CD, noncomplex chronic disease; PMCA, Pediatric Medical Complexity Algorithm.

^a^ A total of 4219 observations were removed owing to missing data.

^b^ Other/unknown race includes Asian, American Indian, Hawaiian or Pacific Islander, multiple races, and unreported.

### Estimated Average PMPM Cost When Disenrollment Is Prevented

The estimated PMPM cost for the full cohort under the natural course scenario in which all costs were included, including costs accrued by children who had coverage gaps, was $125.73 (95% CI, $124.31-$127.15). Estimated PMPM cost for the full cohort in the counterfactual scenario in which disenrollment was prevented was $122.14 (95% CI, $120.64-$123.64). Across subgroups, estimated PMPM costs were $2 to $8 lower in the counterfactual scenario compared with the natural course across all subgroups (Table 3).

**Table 3.** Estimated per Member per Month Cost in Natural Course Scenario Compared With a Counterfactual Scenario in Which Disenrollment Is Prevented

Subgroup	No. (%)	PMPM cost (95% CI), $	
		Natural course	Counterfactual (disenrollment prevented)
Overall	795 985 (100)	125.73 (124.31-127.15)	122.14 (120.64-123.64)
Age, y			
1-5	185 512 (23.3)	118.43 (115.94-120.92)	115.83 (112.55-119.11)
6-11	294 721 (37.0)	125.86 (123.41-128.32)	122.54 (118.23-126.85)
12-17	251 690 (31.6)	137.72 (135.32-140.12)	133.47 (128.29-138.65)
18-20	64 062 (8.0)	91.28 (85.63-96.93)	89.31 (34.37-144.25)
Sex			
Female	395 616 (49.7)	114.98 (113.16-116.80)	112.33 (110.02-114.65)
Male	400 369 (50.3)	136.47 (134.25-138.68)	131.70 (128.18-135.21)
Race			
Black	272 093 (34.2)	106.44 (104.20-108.67)	104.17 (98.79-109.55)
White	355 577 (44.7)	145.92 (143.79-148.05)	139.99 (137.45-142.54)
Other/unknown^a^	168 315 (21.1)	115.96 (112.68-119.23)	113.54 (104.43-122.65)
Ethnicity			
Hispanic	109 531 (13.8)	84.44 (81.69-87.19)	84.42 (74.93-93.91)
Not Hispanic	679 865 (85.4)	130.44 (128.98-131.91)	126.27 (124.54-128.01)
Geography			
Appalachian	132 234 (16.6)	135.08 (131.72-138.44)	130.89 (125.56-136.22)
Urban	588 881 (74.0)	125.02 (123.50-126.54)	121.06 (118.96-123.17)
Rural	203 079 (25.5)	128.50 (125.77-131.23)	126.15 (120.95-131.35)
PMCA			
1 Healthy	522 614 (65.7)	55.40 (54.95-55.84)	55.14 (54.70-55.58)
2 NC-CD	136 721 (17.2)	156.33 (154.73-157.93)	156.21 (155.15-157.26)
3 C-CD	136 650 (17.2)	350.49 (342.90-358.09)	342.22 (330.77-353.67)

Abbreviations: C-CD, complex chronic disease; NC-CD, noncomplex chronic disease; PMCA, Pediatric Medical Complexity Algorithm; PMPM, per member per month.

^a^ Other/unknown race includes Asian, American Indian, Hawaiian or Pacific Islander, multiple races, and unreported.

## Discussion

In this study of Medicaid coverage disruption among children in North Carolina, we found that the risk of unstable enrollment was high, with a substantial fraction of the enrolled population of children disenrolling and reenrolling in Medicaid within less than a year. These findings add to previous estimates of pediatric Medicaid enrollment instability, which range widely from 10% to 50%, although most prior estimates predate ACA implementation.^11,12,13,27,28^ The rates of coverage disruption found in this study suggest that despite the significant progress made in insuring children since the Children’s Health Insurance Program Reauthorization Act of 2009 and the ACA, many eligible children continue to experience preventable insurance coverage gaps. We identified key disparities, with the highest rates of coverage disruption among Latinx children, a population that experiences unique barriers related to language access and immigration policy.

Our findings have important implications for strategies to improve children’s insurance coverage. Most parents are eager to enroll their children in Medicaid if eligible, but confusion about eligibility and the reenrollment process can lead to coverage disruptions.^29^ Importantly, 12-month continuous eligibility provisions, which allow children to remain enrolled in Medicaid for a full year unless the child ages out, moves, or voluntarily withdraws, have been in place in North Carolina for over a decade.^30,31^ However, our results suggest that these policies are not sufficient to ensure that all eligible children remain enrolled. A number of policy levers have been proposed as additional solutions to improve coverage rates. These include (1) presumptive eligibility, under which states may authorize qualified entities to make presumptive eligibility determinations for Medicaid/CHIP, (2) leveraging of electronic data sources across programs to verify eligibility, (3) automatic renewal mechanisms, and (4) specialized navigators to address renewal barriers within communities at highest risk of coverage disruptions.^31,32^ Policies that allow schools or other trusted entities that families regularly interact with to enroll children who appear to be eligible could also play a role in improving coverage gaps and uninsurance. However, community-engaged research with subgroups at highest risk of Medicaid coverage disruption, including Latinx families, communities of racial and ethnic minorities, and families with the youngest children, is critical for understanding specific barriers to maintaining enrollment and implementing effective and equitable interventions.

In addition to examining factors associated with pediatric coverage disruption, we applied an innovative approach using inverse probability of censoring weights to estimate the outcome of a hypothetical intervention to prevent Medicaid disenrollment with average medical expenditures in our study population. Pre-ACA analyses show that monthly Medicaid expenditures decrease modestly as children are enrolled for longer periods.^20^ Additionally, policies that reduce pediatric coverage disruption, such as 12-month continuous eligibility provisions, lead to improved retention among eligible beneficiaries and subsequent decreases in short-term care utilization and cost.^8^ Therefore, we hypothesized that preventing disenrollment in the study population would be associated with decreased cost, presumably through improved access to preventive care and decreased utilization of higher-cost short-term care. We found that preventing Medicaid disenrollment was associated with a small decrease in estimated monthly costs. However, cost was a strong driver of enrollment in these analyses, suggesting that families who anticipate that children may need higher-cost care or more engagement with the health care system are less likely to allow coverage disruptions. Therefore, some portion of the reduced cost seen in the counterfactual scenario in which disenrollment is prevented could be due to retaining healthier and less costly patients, rather than only decreasing costs in patients who experience coverage disruptions. Reductions in PMPM cost seen when preventing disenrollment may not offset the cost of additional member months—for example, if continuous eligibility provisions were put in place. However, preventing insurance coverage gaps during childhood could confer longer-term savings that could not be assessed over the 36-month study period.

### Limitations

Our results are subject to several limitations in addition to those discussed previously. We could not distinguish children who experienced coverage gaps from children who remained disenrolled. If children who remained disenrolled incurred lower cost owing to unmeasured factors, such as gaining other coverage, being healthier, or not needing emergent care, then the difference in cost found here could underestimate the difference in cost specific to preventing coverage disruption. To further understand the outcome of coverage disruption on cost outcomes, additional research is needed to identify key subgroups that drive utilization and cost disparities after experiencing coverage gaps. Small clinical subgroups of children with medical complexity may be of particular importance in driving cost increases after experiencing coverage disruptions. For example, among adults, short-term care utilization among all Medicaid-covered adults does not increase substantially with coverage gaps.^33^ However, coverage gaps for adults with severe depression nationally led to increased utilization, with cost increases of more than $300 PMPM.^34^ Additionally, the use of claims data precluded us from understanding reasons for disenrollment or insurance coverage outside of Medicaid. The generalizability of this single-state study may also be limited in the context of state-level variations in Medicaid policy.

## Conclusions

Despite improvement in Medicaid retention rates among children over the past decade, in this cohort study of children enrolled in North Carolina Medicaid from 2016 to 2018, many children continued to experience preventable insurance coverage gaps. Medicaid coverage disruption is particularly prevalent among Latinx children, a population already at increased risk for health disparities and social risk.^35^ These findings have key implications to inform the transition to Medicaid managed care in North Carolina, as policy makers seek to implement strategies to reduce coverage disruptions during this transition. Additional policy initiatives are needed to address persistent insurance discontinuity in Medicaid and ensure that all children have equitable, continuous access to health care.

## Notes

Supplement. **eAppendix.** Statistical methodology
