# Supplementary material for: Medicaid Coverage Disruptions Among Children Enrolled in North Carolina Medicaid From 2016 to 2018
Source: JAMA Health Forum. 2021 Dec 23;2(12):e214283. doi: 10.1001/jamahealthforum.2021.4283 (PMC8796937; doi:10.1001/jamahealthforum.2021.4283)
Supplement: Supplement. — eAppendix. Statistical methodology [file jamahealthforum-e214283-s001.pdf]

Supplemental Online Content

Cholera R, Anderson D, Raman SR, et al. Medicaid coverage disruptions among children enrolled in North Carolina Medicaid from 2016 to 2018. *JAMA Health Forum*. 2021;2(12):e214283. doi:10.1001/jamahealthforum.2021.4283

eAppendix. Statistical methodology

This supplemental material has been provided by the authors to give readers additional information about their work.

© 2021 Cholera R et al. *JAMA Health Forum.*

eAppendix. Statistical methodology

In the cost analysis we sought to estimate the impact of Medicaid churn on average per member per month (PMPM) costs. We assessed the average per member per month costs over the study period. We then separately censored individuals who disenrolled and re-estimated the average costs in an inverse probability of censoring weighted population.

The per member per month (PMPM) cost was estimated under three scenarios:

1. **Natural course estimator:**

   The natural course estimator includes all actual costs accrued across any period of enrollment. The estimator first computes the average cost in each month (for 36 months). The estimated value is the average of these average monthly costs. PMPM cost under the natural course was estimated with:

   $$\hat{E}[C] = \frac{1}{m}\sum_{i=1}^{m}\frac{1}{n_m}\sum_{j=1}^{n_m} C_{i,m}\delta_{i,m}$$

   where m is the number of weeks over which to estimate costs, C_m is the costs accrued during week m, n_m is the number of individuals enrolled in week m, and δ_m is an indicator that an individual was enrolled during month m.

   Patients are only included in the natural course when they were enrolled in Medicaid. The natural course estimator should therefore *reflect the high costs seen for patients who disenrolled and selectively re-enrolled* during months of poor health and expensive care.

2. **Unadjusted estimator**: The unadjusted estimator only includes costs up to the first disenrollment. This means that for patients who remain enrolled the entire time, all their costs are included. However, for those who experience any disenrollment, any costs accrued during subsequent periods of reenrollment are discarded for the unadjusted estimator. The estimator first computes the average cost in each month (36 months). The estimated value is the average of these average monthly costs.

3. **Counterfactual estimator:** The counterfactual estimator includes costs up to the first disenrollment, i.e. the same costs as the unadjusted estimator. This means that for patients who remain enrolled the entire time, all their costs are included. However, for those who experienced any disenrollment, *any costs accrued during subsequent periods of re-enrollment were discarded*. The counterfactual estimator then used a censored cost estimator to provide an estimate of the counterfactual PMPM if no disenrollment occurred. This method, adapted from Bang and Tsiatis 2000, uses inverse probability of censoring weights (IPCW) to estimate the counterfactual costs if no disenrollment occurred:

$$\hat{E}[C^*] = \frac{1}{m}\sum_{i=1}^{m}\frac{1}{n}\sum_{j=1}^{n}\frac{C_{i,m}\delta_{i,m}}{\widehat{\Pr}(\delta_{i,m} = 1 | W_i)}$$

where W is a vector of covariates sufficient to achieve conditional exchangeability between those who are censored due to disenrollment and those who are uncensored. The weights were estimated using a logistic regression model with a set of covariates sufficient to achieve conditional exchangeability between those who are censored due to disenrollment and those who are uncensored. In short, this estimator is weighted in such a way as to estimate the PMPM cost in the counterfactual setting where disenrollment is prevented.

The counterfactual estimator should reflect the PMPM costs that would accrue over the full 36 month time period if patients who disenrolled under natural course had been prevented from disenrolling altogether (with the conditional exchangeability assumptions as above). The counterfactual estimator explicitly accounts for the smaller denominator by upweighting the remaining people to stand in for the people who are censored for disenrollment. Since the weights make these beneficiaries look like those who disenrolled, we are able to, under strong assumptions, interpret the results as what would happen if nobody disenrolled. The denominator increases due to the weights, but so does the numerator.

Under the hypothesis that patients who churn might impact cost through selective enrollment during expensive healthcare episodes, we would expect such higher costs to be reflected in the natural course scenario but not in the counterfactual scenario. This comparison of counterfactual and natural course estimators is the comparison of interest.

Model details

All costs were aggregated to the level of week before modelling.

For the total estimates of all individuals in a given cohort, the logistic regression model used to estimate the weights modeled the disenrollment outcome with the following covariates: week, baseline age, lagged average weekly cost, and cost accrued in the previous month. The model also included the categorical PMCA level.

Categorical variables were modeled with binary dummy variables. The continuous variable week was modeled with a restricted cubic regression spline with 5 knots to allow a very flexible model for the time trend. The continuous variables lagged average weekly cost and cost accrued in the past month were modeled with restricted cubic regression splines with 3 knots.

© 2021 Cholera R et al. *JAMA Health Forum.*
